# Brown bear attacks on humans: a worldwide perspective

**DOI:** 10.1038/s41598-019-44341-w

**Published:** 2019-06-12

**Authors:** G. Bombieri, J. Naves, V. Penteriani, N. Selva, A. Fernández-Gil, J. V. López-Bao, H. Ambarli, C. Bautista, T. Bespalova, V. Bobrov, V. Bolshakov, S. Bondarchuk, J. J. Camarra, S. Chiriac, P. Ciucci, A. Dutsov, I. Dykyy, J. M. Fedriani, A. García-Rodríguez, P. J. Garrote, S. Gashev, C. Groff, B. Gutleb, M. Haring, S. Härkönen, D. Huber, M. Kaboli, Y. Kalinkin, A. A. Karamanlidis, V. Karpin, V. Kastrikin, L. Khlyap, P. Khoetsky, I. Kojola, Y. Kozlow, A. Korolev, N. Korytin, V. Kozsheechkin, M. Krofel, J. Kurhinen, I. Kuznetsova, E. Larin, A. Levykh, V. Mamontov, P. Männil, D. Melovski, Y. Mertzanis, A. Meydus, A. Mohammadi, H. Norberg, S. Palazón, L. M. Pătrașcu, K. Pavlova, P. Pedrini, P. Y. Quenette, E. Revilla, R. Rigg, Y. Rozhkov, L. F. Russo, A. Rykov, L. Saburova, V. Sahlén, A. P. Saveljev, I. V. Seryodkin, A. Shelekhov, A. Shishikin, M. Shkvyria, V. Sidorovich, V. Sopin, O. Støen, J. Stofik, J. E. Swenson, D. Tirski, A. Vasin, P. Wabakken, L. Yarushina, T. Zwijacz-Kozica, M. M. Delgado

**Affiliations:** 1 Research Unit of Biodiversity (UMIB, UO-CSIC-PA), Oviedo University - Campus Mieres, Mieres, Spain; 2 Museo delle Scienze, Sezione Zoologia dei Vertebrati, Corso del Lavoro e della Scienza 3, I-38123 Trento, Italy; 3 Department of Conservation Biology, Estación Biológica de Doñana-CSIC, Calle Américo Vespucio s/n, E-41092 Sevilla, Spain; 4 Instituto Pirenaico de Ecología, C.S.I.C., Avda. Nuestra Señora de la Victoria 16, 22700 Jaca, Spain; 5 Institute of Nature Conservation, Polish Academy of Sciences, Warsaw, Poland; 6 Department of Wildlife Ecology and Management, Faculty of Forestry, Düzce University, Düzce, Turkey; 7 Kondinskie Lakes National Park named after L. F. Stashkevich, Sovietsky, Russian Federation; 8 A. N. Severtsov Institute of Ecology and Evolution, Russian Academy of Sciences, Moscow, Russian Federation; 9 Institute of Plant and Animal Ecology, Ural Branch, Russian Academy of Sciences, Moscow, Russian Federation; 10 Sikhote-Alin State Nature Biosphere Reserve named after K.G. Abramov, Pinezhsky, Russian Federation; 11 Office National de la Chasse et de la Faune Sauvage, Besançon, France; 12 LIFEURSUS Project, Environmental Protection Agency, Voluntary, Romania; 13 Department of Biology and Biotechnologies, La Sapienza University of Rome, Rome, Italy; 14 Balkani Wildlife Society, Sofia, Bulgaria; 15 Department of Zoology, Ivan Franko National University of Lviv, Lviv, Ukraine; 16 Centre for Applied Ecology “Prof. Baeta Neves”/InBIO, Institute of Agronomy, University of Lisbon, Lisboa, Portugal; 17 Tyumen State University, Tyumen, Russian Federation; 18 Forest and Wildlife Service, Provincia Autonoma Trento, Trento, Italy; 19 Nature Conservation, Government of Carinthia, Carinthia, Austria; 20 Slovak Wildlife Society, Liptovský Hrádok, Slovakia; 21 Finnish Wildlife Agency, Helsinki, Finland; 22 Department of Biology, University of Zagreb, Zagreb, Croatia; 23 Department of Environmental Science, Faculty of Natural Resources, University of Tehran, P.O. Box: 4111, 31587-77871 Karaj, Iran; 24 Altai State Nature Biosphere Reserve, Barnaul, Russian Federation; 25 ARCTUROS, Civil Society for the Protection and Management of Wildlife and the Natural Environment, 53075 Aetos, Florina Greece; 26 Forest Research Institute Karelian Research Centre Russian Academy of Sciences, Petrozavodsk, Russian Federation; 27 Hingansky, Moscow, Russian Federation; 28 Lviv Forestry and Wood-Technology University, Lviv, Ukraine; 29 Natural Resources Institute, Rovaniemi, Finland; 30 Department of Animal Ecology, Russian Research Institute of Game Management and Fur Farming, 79 Preobrazhenskaya Str., Kirov, 610000 Russia; 31 Institute of Biology, Komi Science Centre, Russian Academy of Sciences, Petrozavodsk, Russian Federation; 32 State Nature Reserve Stolby, Krasnoyarsk, Russian Federation; 33 Biotechnical Faculty, Department of Forestry, University of Ljubljana, Ljubljana, Slovenia; 34 University of Helsinki, Helsinki, Finland; 35 Federal Center for Integrated Arctic Research, Russian Academy of Sciences, Moscow, Russian Federation; 36 Estonian Environment Agency, Tallinn, Estonia; 37 Macedonian Ecological Society, Skopje, Macedonia; 38 Department of Wildlife Sciences, University of Göttingen, Göttingen, Germany; 39 CALLISTO Wildlife and Nature Conservation Society, Vasilikós, Greece; 40 The State Natural Reserve Tungusky, Krasnoyarsk State Pedagogical University VP Astafieva, Krasnoyarsk, Russian Federation; 41 Department of Environment Sciences, Faculty of Natural Resources, University of Jiroft, Jiroft, Iran; 42 Finnish Wildlife Agency, Helsinki, Finland; 43 Territory and Sustainability Department, Generalitat of Catalonia, Barcelona, Spain; 44 Association for the Biological Diversity Conservation, Focşani, Romania; 45 FSBI “Zeya State Nature Reserve”, Zeya, Russian Federation; 46 State Nature Reserve Olekminsky, 678100, Rebublic Sakha, Olekminsk Filatova 6, Russian Federation; 47 Pinezhsky State Nature Reserve, Pinezhsky, Russian Federation; 48 Wildlife Section, Norwegian Environment Agency, Trondheim, Norway; 49 Pacific Geographical Institute, Russian Academy of Sciences, FEB RAS, 7 Radio St., Vladivostok, Russian Federation; 50 Far Eastern Federal University, 8 Sukhanova St., Vladivostok, Russian Federation; 51 Freelance Researcher, Moscow, Russian Federation; 52 V. N. Sukachev Institute of Forest SB, Russian Academy of Sciences, Krasnoyarsk, Russian Federation; 53 Department of Scientific Research and International Collaboration of Kyiv Zoo, Kiev, Ukraine; 54 Institute of Zoology, National Academy of Science, Minsk, Belarus; 55 Norwegian Institute for Nature Research, Trondheim, Norway; 56 Faculty of Environmental Sciences and Natural Resource Management, Norwegian University of Life Sciences, Ås, Norway; 57 Poloniny National Park, Snina, Poland; 58 State Nature Reserve Malaya Sosva, Sovetsky, Russian Federation; 59 Faculty of Applied Ecology and Agricultural Sciences, Hedmark University College, Elverum, Norway; 60 Tatra National Park, Zakopane, Poland

**Keywords:** Behavioural ecology, Conservation biology

## Abstract

The increasing trend of large carnivore attacks on humans not only raises human safety concerns but may also undermine large carnivore conservation efforts. Although rare, attacks by brown bears *Ursus arctos* are also on the rise and, although several studies have addressed this issue at local scales, information is lacking on a worldwide scale. Here, we investigated brown bear attacks (n = 664) on humans between 2000 and 2015 across most of the range inhabited by the species: North America (n = 183), Europe (n = 291), and East (n = 190). When the attacks occurred, half of the people were engaged in leisure activities and the main scenario was an encounter with a female with cubs. Attacks have increased significantly over time and were more frequent at high bear and low human population densities. There was no significant difference in the number of attacks between continents or between countries with different hunting practices. Understanding global patterns of bear attacks can help reduce dangerous encounters and, consequently, is crucial for informing wildlife managers and the public about appropriate measures to reduce this kind of conflicts in bear country.

## Introduction

Large carnivore attacks on humans are the most dramatic form of human-wildlife conflicts^1,2^. Although rare compared to attacks by other wildlife and domestic species^3,4^, such incidents are on the rise in many areas around the world^2,5–7^. Such a trend not only raises human safety concerns, but also undermines large carnivore conservation efforts, as well as the recovery of several of these species around the world^8,9^. Indeed, when they do occur, attacks on humans elicit considerable media attention, which can lead people to overestimate the risk of an attack and, eventually, cause negative public reactions and opposition towards conservation actions^10–12^. Additionally, when using negative framing and graphic contents to describe an attack, the media does not help to correctly inform people on how to avoid encountering large carnivores and how to behave in case of an encounter, but it rather unnecessary alarms the public about a phenomenon that is actually very rare^2,12^. As mentioned in previous studies^2,13,14^, by providing citizens with an objective description of the events and correct information on how to avoid conflicts, the media has the power to promote both human safety and carnivore conservation^12^. Because one of the most important ways to minimize this type of conflict is to gain a deeper understanding of the circumstances triggering large carnivore attacks, as well as of potential factors associated with such incidents^2,14^, it is extremely important to provide managers and the public with accurate and objective knowledge to reduce their occurrence.

The end of legal and widespread persecution, strict protection measures, and reintroductions have allowed brown bear *Ursus arctos* populations to recover and expand in many areas of North America and Europe^9,15–18^. Currently, brown bears are estimated to exceed 200,000 individuals worldwide, most of which live in Russia (~100,000), whereas North America and Europe are home to around 58,000 and 15,400 brown bears, respectively^16^. Although the number of bears is growing globally, several small subpopulations are still endangered and, in several cases, their location in close proximity to highly humanized areas leads to increased negative interactions with humans^16^.

Although brown bears are known to adjust their behaviour in order to avoid humans^19^, complete avoidance is not always possible. Brown bears, indeed, are known to be involved in various types of conflicts with humans, which typically include property damage^15^ and, more rarely, direct attacks on people^2^. These conflicts might be even more severe in areas of recent expansion and reintroduction, where bears had previously been extirpated. Here, traditional prevention practices have been lost and people are no longer used to sharing the landscape with this large carnivore^15,20^. At the same time, human population is growing all around the world, leading to an expansion of urban areas towards natural habitats^21^. In addition, in developed countries, people living in cities are increasingly engaged in recreational outdoor activities in natural parks^2^, and owning a second house in natural areas outside the city has become a common trend^22^. Such intensified use of wilderness area by humans, especially people that are not used to cohabit with wildlife, increases the probabilities of potentially dangerous encounters with these species, urging wildlife managers and conservationists to take action.

Among all large terrestrial and aquatic predators, attacks by brown bears are the most highly covered by the international media^12^. This suggests that, even if attacks by brown bears are less frequent than those by other predators, at least among North American large carnivores^2^, this species has the power to attract amplified attention of mass media, which has the potential to negatively impact public attitude.

Several studies have investigated attacks on humans by brown bears at local scales, suggesting a general increase in the number of incidents over the years in different regions of the world (e.g.)^1,14,21–24^. However, most of the published literature on the topic is concentrated in North America and Scandinavia, and large-scale studies are lacking.

Here, we investigated patterns of brown bear attacks on humans occurring between 2000 and 2015 on a worldwide scale, with the main aim of improving our knowledge on this type of conflict and, consequently, providing useful information that could help reduce the occurrence of negative human-bear encounters. In particular, we: (*i*) provide a first global-scale perspective of the phenomenon; (*ii*) describe temporal and spatial patterns of these incidents; (*iii*) describe main attack circumstances, highlighting common features and local peculiarities in attack scenarios between geographical areas with different histories of human coexistence with this species (e.g. North America *vs*. Europe); and (*iv*) explore the effect of various factors, such as bear and human densities, as well as differences in geographic location and management practices, on the number of attacks. In this regard, we hypothesised that:

(*a*) higher numbers of attacks occurred in those countries/jurisdictions where both bear and human densities are higher, due to the consequent higher encounter probability; and (*b*) fewer attacks occurred in those countries where bears are legally hunted, due to potential removal of bold individuals.

## Results

### Spatio-temporal patterns of the attacks

**Figure 1:**
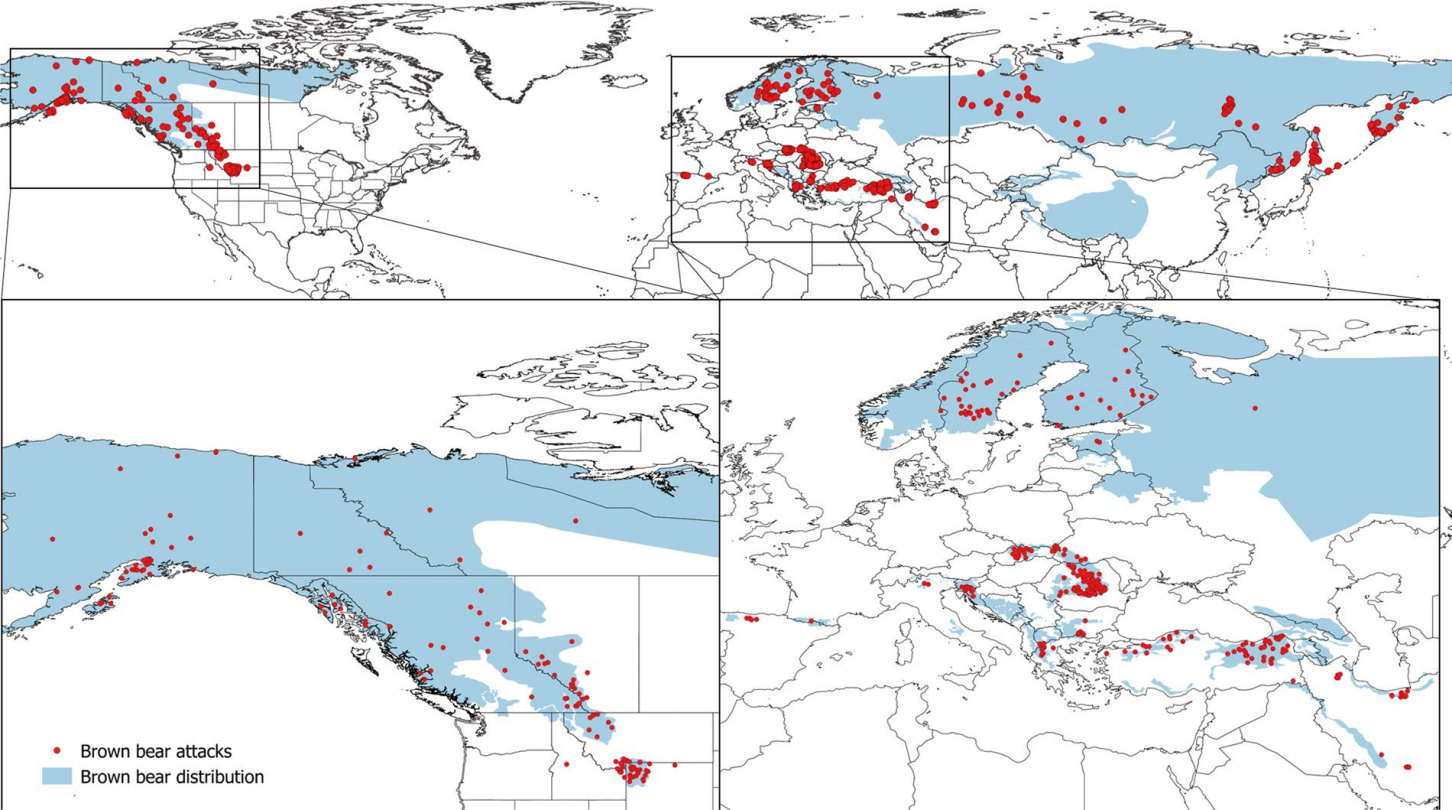
Spatial distribution of brown bear attacks on humans recorded between 2000 and 2015. Only attacks for which at least approximate coordinates were available are shown in the map (95% of all the attacks recorded, n = 634). In some cases, one point corresponds to more than one attack. The map was created in QGIS software^51^, using the world borders layer^59^ and the IUCN layer of brown bear distribution, including both permanent and occasional presence^53^.

Our search resulted in a total of 664 attacks between 2000 and 2015 from the three main geographical blocks of the brown bear distribution: West (i.e. and hereafter North America, n = 183), Centre (i.e. and hereafter Europe, n = 291), and East (i.e. Russia, Iran and Turkey, hereafter East, n = 190), for which at least information regarding the year was available (Fig. 1 and Table 1). We also recorded an additional 61 cases of attacks from the published literature (4 cases in Albania, 11 in Bosnia and Herzegovina, 9 in Macedonia, 2 in Nepal and 35 in Japan), which we did not include in the analyses due to the lack of sufficient information. We recorded an attack rate of 39.6 attacks/year globally: 11.4 attacks/year in North America and 18.2 attacks/year in Europe (10 attacks/year, if we exclude Romania). The recorded value of 19 attacks/year in the East probably represents an underestimation, due to the lack of information for several regions of the continent. Most attacks, 85.7% (n = 568), resulted in human injury and 14.3% (n = 95) ended with the death of the person involved. Specifically, 19 deaths occurred in Europe (6.6% of the attacks recorded in Europe), 24 in North America (13.1% of the total attacks in North America) and 52 in the East (32.0% of the total attacks in the East).

**Table 1:** Number of brown bear attacks on humans recorded during the period 2000–2015 and characteristics of the country/jurisdiction where the attacks occurred.

Country/State	Number of Attacks (2000–2015)	Number of Fatalities (2000–2015)	Number of Brown Bears	Brown Bear Range (km^2^)	Human Density (inhabitants/km^2^)	Brown Bear Density (bears/1000 km^2^)
Romania	131	11	6000	89900	62.3	66.741
Slovakia	54	0	1000	12855	89.0	77.790
Turkey	54	11	4000	190552	29.7	20.992
Alaska	51	7	32000	1455855	0.3	21.980
British Columbia	42	2	15000	768801	0.4	19.511
Wyoming	29	5	511	27896	1.2	18.318
Sweden	28	2	2900	316300	5.0	9.169
Iran	25	0	unknown	241327	12.7	unknown
Montana	25	2	1105	64713	2.9	17.075
Alberta	18	4	691	148114	0.8	4.665
Finland	17	0	1700	357900	13.7	4.750
Greece	12	1	350	19500	26.8	17.949
Slovenia	12	0	455	13700	73.3	33.212
Poland	8	1	115	10400	75.7	11.058
Ukraine	8	2	350	28000	101.0	12.500
Idaho	8	0	34	6663	3.5	5.103
Bulgaria	7	1	560	32800	35.2	17.073
NW Territories	6	1	4000	772227	0.01	5.180
Spain	5	0	247	12800	7.2	19.297
Yukon	4	3	6000	480406	0.03	12.489
Croatia	3	0	1000	12372	21.5	80.828
Norway	2	0	105	149550	6.9	0.702
Italy (Alps)	2	0	51	2000	92.4	25.500
Estonia	2	0	700	34000	19.2	20.600

Values are calculated within the brown bear population of each country where the attacks took place. When attacks occurred in more than one bear population within one country, values were calculated for the total area occupied by the populations involved.

Within Europe, most attacks occurred in Romania (n = 131), followed by Slovakia (n = 54), Sweden (n = 28), and Finland (n = 17). In North America, most of the attacks occurred in Alaska (n = 51), British Columbia (n = 42), Wyoming (n = 29), Montana (n = 25), and Alberta (n = 18). In the East, we recorded 111 attacks in Russia, 25 in Iran and 54 in Turkey (Fig. 1 and Table 1).

**Figure 2:**
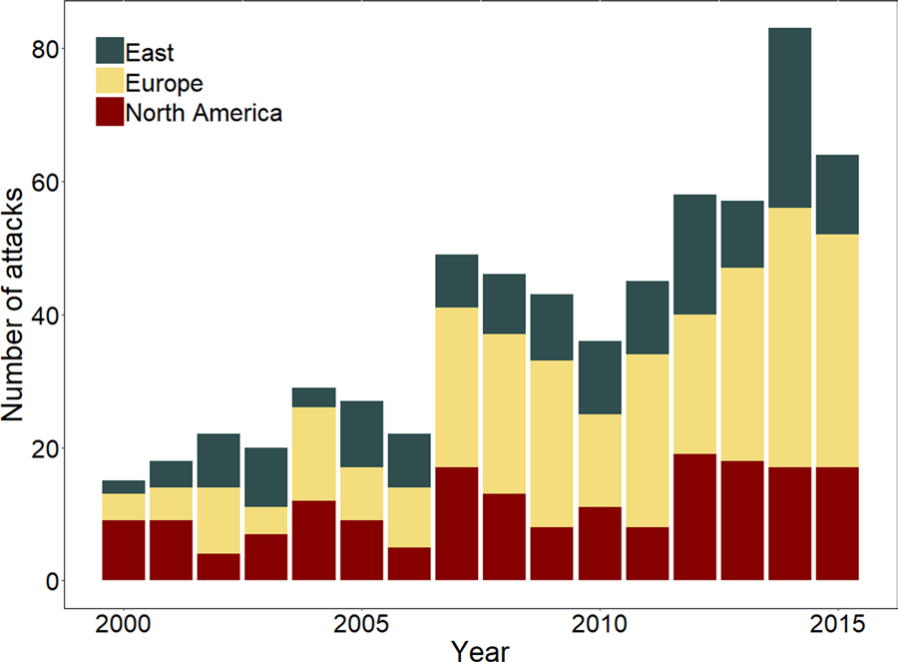
Temporal trends of reported brown bear attacks on humans throughout the species’ range during 2000–2015, n = 664.

The number of attacks increased worldwide over the years (Fig. 2; Supplementary Tables S1 and S2; Supplementary Fig. S1), with most of the attacks occurring in summer (48%; Supplementary Fig. S2a) and during daytime (73%; Supplementary Fig. S2b).

### Main circumstances of the attacks

**Figure 3:**
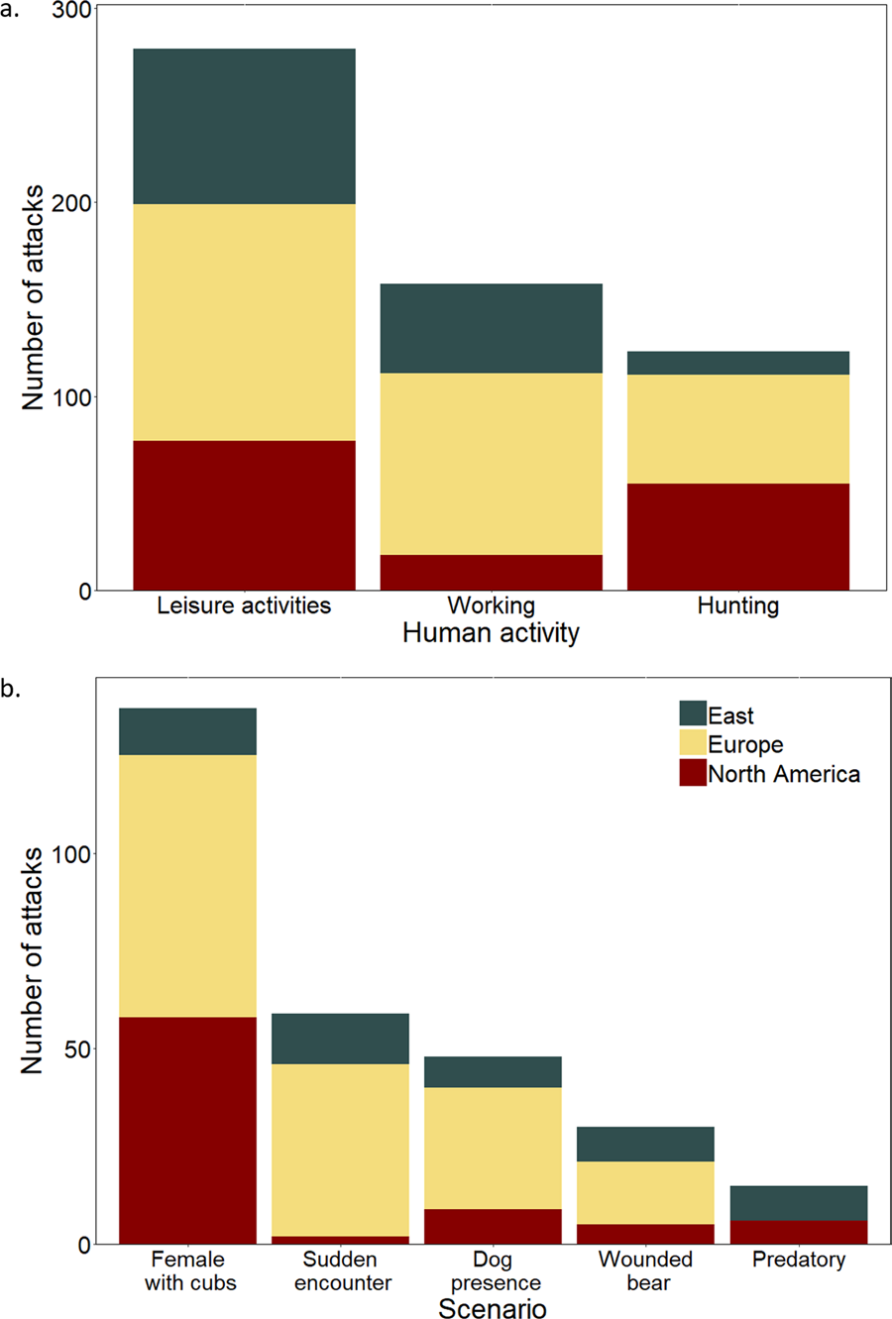
(**a**) Main activities carried out by people at the moment of an attack (n = 560). (**b**) Main scenarios of the attacks (n = 289).

Attacked people were almost exclusively adults (99%; Supplementary Fig. S3a) and males (88%; Supplementary Fig. S3b). In 63% of the cases, the person was alone at the moment of the attack (Supplementary Fig. S3c). When the attack occurred, 50% (n = 279) of the people were engaged in leisure activities, such as hiking (n = 88), picking berries, mushrooms, or antlers (n = 64), camping (n = 31), fishing (n = 18), or jogging (n = 17). As for the other activities, 28% (n = 158) of the attacked people were working outside, i.e. farming, guarding livestock, or logging (n = 104), or doing wildlife-related fieldwork (n = 12), and 22% (n = 123) were hunting (Fig. 3a). Attacks that occurred during bear hunts (n = 27) were concentrated in a few countries/states (Sweden, Finland, Alaska and Russia). In Europe, this kind of attack was only present in Fennoscandia (n = 16), where bears are often hunted with chasing dogs. No attacks occurred in European countries where bears are hunted using bait from a stand. The attacks that occurred while working outside were more frequent in Europe (n = 94, of which 64 occurred in Romania) than in the rest of the brown bear range included in our study.

**Figure 4:**
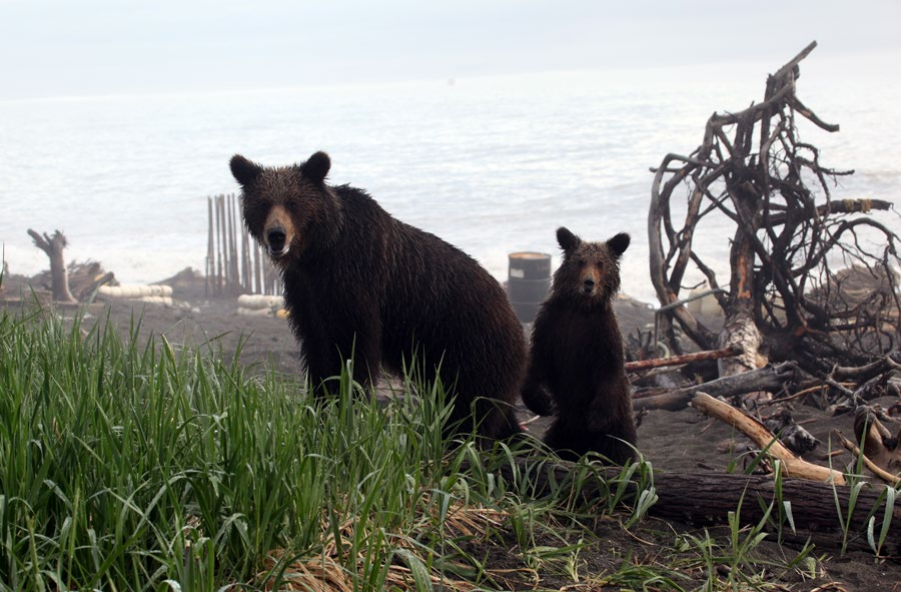
Nearly half (47%) of the attacks recorded worldwide were the result of a defensive reaction of a female with cubs. Photo by Ivan Seryodkin.

The most prevalent scenario of a brown bear attack was an encounter with a female bear with cubs (47%, n = 137; Fig. 4), followed by sudden encounters (20%, n = 59), dog presence (17%, n = 48), bear attacking after being shot or trapped (10%, n = 30), and predatory attacks (5%; n = 9 in Russia and n = 6 in North America) (Fig. 3b). However, sometimes the scenario was more complex, because an attack could have been triggered by more than one factor. For example, in seven cases, the attack was caused by the interaction of a female with cubs and a dog.

### Correlates of the attacks

We explored general spatio-temporal patterns of bear attacks and found that the following four factors were the most important in explaining the number of attacks: (a) year of the attack, (b) bear density, and (c) human population density (Supplementary Tables S1 and S2). In such analysis, the Eastern part of bear distribution was excluded, given the spatial heterogeneity of the data, as we were able to only collect attack data from Iran, Turkey and some Russian areas. The importance of these three variables was also confirmed by their P-values (P always ≤0.01) and confidence intervals (Supplementary Table S2). Specifically, we observed: (1) a significant increase in the number of attacks over the years; (2) higher bear density was associated with higher number of recorded attacks; whereas (3) attacks were negatively correlated with human population density. Bear density explained the largest proportion of variance (Supplementary Table S2). We found no significant difference in the number of attacks between continents or between countries with different management practices.

## Discussion

This first large-scale overview of brown bear attacks on humans has highlighted several elements that may serve as a general framework to this worldwide issue. Our results show a global increase in the number of attacks over the last decades, which is likely the result of several factors, such as the growth of both bear and human populations worldwide, that has led to increased habitat overlap. Additionally, a growing number of people is engaged in recreational activities in bear areas, which likely enhances the probability of encounters^2^. Seasonal and circadian patterns were similar between continents. Europe had a slightly higher number of attacks during winter than North America and the East, which might be because hibernation is usually shorter in Europe^25^ and, thus, brown bears are more active in Europe during this season than on the other continents. The peak of attacks during the summer and during daylight is likely due to the fact that humans mainly engage in recreational activities or outdoor work in bear habitats during this season and are generally most active during the day. These patterns are in line with previous studies on attacks by brown bears both in North America^2,14^ and Scandinavia^1^. Most people were engaged in leisure activities at the moment of the attack. This suggests that attacks mainly occur when people visit bear areas for recreational purposes, which is especially true in North America, where only a few attacks occurred to people working outside.

The fact that attacked people were almost exclusively adults is in line with the main human activities and attack scenarios. Indeed, when attacked by large carnivores, children are usually involved in predatory attacks, which are generally very rare for brown bears^26^. The fact that the most prevalent scenario, both in North America and Europe, was an encounter with a female with cubs agrees with that found in previous studies at smaller scales^1,14^ and suggests that this class of bear is more likely to respond aggressively to encounters with humans and, therefore, requires additional attention and public information campaigns. Additionally, females with cubs, together with subadults, are most likely to use areas close to human activities in order to avoid male bears and predators or search for anthropogenic food^27–30^, and this might make them more susceptible to accidental encounters with people. In this sense, where possible, temporal restrictions on public access to areas where females with cubs are commonly present might be crucial for avoiding human disturbance to brown bear females and resulting dangerous encounters. Other frequent scenarios (sudden encounters, presence of dogs, wounded bear) are mainly the result of inappropriate and risk-enhancing human behaviours (e.g. moving alone and being silent in bear country, walking an unleashed dog, or chasing a wounded bear while hunting), and could be reduced by improving public education and awareness of the issue^2^. For example, when in bear country, announcing one’s presence can help to avoid sudden encounters and unleashed dogs should be strongly avoided. In Alaska, dogs have been found to help terminate attacks in nearly half of the cases^14^; however, in other cases both during leisure activities and hunting, dogs are known to have initiated the attack^1,2,14^. Therefore, keeping them leashed when engaging in recreational activities might reduce the occurrence of such incidents, while it might still help to deter the attack, when it happens. Additional preventive measures such as proper garbage management, securing trash and food containers both in developed and natural areas frequented by tourists can help prevent bears from approaching people and cause conflicts^31,32^.

The main profile of the involved humans and the main scenarios agree with what has been highlighted in previous studies. In Alaska, for instance, Smith and Herrero^14^ found that the majority of people involved in attacks by bears were adult males (83%), who were primarily engaged in hiking and hunting. The authors also found that interactions were usually initiated by humans (59%), such as people surprising bears, wounding them in hunts, or disturbing them while defending a carcass^14^. In the same study, unaccompanied individuals were also observed to be more vulnerable to attacks than people in groups, which is in line with our findings, as well as findings for other bear species in North America^33,34^. The reason for this is likely related to the fact that larger groups are more easily detectable and, consequently, avoided by bears, because groups tend to make more noise than a lone person. Additionally, when an encounter actually occurs, bears are more likely to flee than attack when confronted with a group of people^14^. The above-mentioned, as well as other, basic precautions are key to reducing the occurrence of dangerous encounters and should be constantly made available to people entering bear areas. For instance, in some regions of North America, warning panels are placed at trails’ entrance, on which basic safety measures are provided. Additionally, visitors of National Parks receive a mandatory presentation and safety instructions on how to behave in bear country. In Russia, which hosts the largest number of brown bears and has the highest recorded number of attacks on humans, specialized manuals were developed, published, and distributed in large quantities to minimize conflicts with bears (e.g.^35,36^). Bear spray is also commonly used in North America as a personal safety measure by people who engage in recreation and work in bear areas, and its effectiveness in deterring attacking bears has been demonstrated^14,37^. This non-lethal deterrent has also been successfully used in Slovakia (R. Rigg, unpublished data), however, it is currently illegal in many other European countries. Such evidence highlights the need for further investigation on its effectiveness and potential implementation in other countries with growing number of bears.

Although we did not find any difference in the number of attacks between continents, there was a remarkable difference in the number of attacks among European countries. Indeed, Romania accounted for nearly half (45%) of all attacks registered in Europe. Romania is home to the largest number of bears in Europe and traditional semi-subsistence agriculture and livestock husbandry are still common in the Carpathian Mountains^38^. In particular, sheep and goats, which are the most vulnerable livestock to bear depredation^38^, are the most common and are increasing year to year^39^. This might explain the fact that 50% of bear attacks in Romania for which detailed information was available (n = 109) involved shepherds, farmers, or cattle herders. Interestingly, in at least 8 cases, the person was attacked while chasing or directly harassing the bear after it had attacked livestock or crops. We therefore suggest that education campaigns on how to avoid or react to close encounters with brown bears and improved livestock protection measures should particularly target Romanian shepherds, farmers, and cattle herders. This scenario differs vastly from the general patterns observed in other brown bear countries, where attacks mainly occurred during leisure activities. These differences in attack patterns suggest that the occurrence of attacks might also greatly depend on local socio-cultural contexts and human behaviours^2^.

Previous studies analysing brown bear attacks at a local scale have shown a correlation between the number of attacks and the growth of the human population at a national or continental scale^14,34^. These studies suggest that the more people live and work in bear areas, the greater the probability of an encounter with this species^14^. Another study found a relationship between the number of attacks on hunters and the brown bear harvest in Scandinavia^1^, but there was no clear relationship with the number of attacks on people involved in non-hunting outdoor activities and the brown bear population size. However, at a global scale, attacks were more frequent in those countries/jurisdictions where human density is lower and bear density higher. Because human density is a measure of the degree of human encroachment into bear range, our results suggest that attacks are less frequent where human developments and activities extend more into bear areas, and more frequent in countries where recreational activities in bear areas are more common. This result might also suggest that bears and people have learnt to coexist better in highly humanized regions, whereas those people who are more at risk of attack are visitors of high bear-density areas, where bears are less accustomed to encountering people, because of lower human density and, consequently, bears and people might be less used to avoiding each other. Additionally, there was no significant difference in the number of attacks between ‘hunting’ and ‘non-hunting’ countries, which does not support our initial hypothesis that fewer attacks occurred in countries where bears are legally hunted.

To conclude, negative encounters with brown bears are extremely rare and mainly non-fatal. However, to increase both human and bear safety, and promote coexistence, it is crucial to gain a deeper understanding, and promote public knowledge of the riskiest circumstances that may trigger an aggressive response by brown bears. To this aim, strong connection and collaboration between researchers, managers and education tools such as mass media and schools should be established to promote correct and scientific-based information about bears among the large public. This first worldwide approach showed that, although similar patterns in attacks exist across the distribution range of brown bears, specific local contexts might prove to be crucial in explaining particularly high or low attack numbers. We therefore believe that, although it is important to have a global picture, additional studies at a local scale, especially in those countries where information is still scarce, will help identify additional factors related to local situations which will provide wildlife managers with specific information on how to effectively deal with this issue.

## Methods

### Collection of information on brown bear attacks

#### Brown bear attack data

We collected reports of brown bear attacks on humans that resulted in physical injury (i.e., the person required medical attention) or death from 2000 to 2015 all around the world. We limited our search to attack cases occurred starting from 2000 as, before that year, information on attacks was scarce. Attack records were collected from personal datasets of the co-authors, published literature^40^, unpublished reports, PhD/MS theses, webpages, and news reports. We searched for the above-mentioned sources using the search engines Google^41^ and Google Scholar^42^. In particular, to collect cases in North America, in addition to these sources, we also carried out a systematic search of news articles on Google for each jurisdiction on an annual basis, using the combination of the following terms: ‘brown bear’ or ‘grizzly’ + ‘attack’ or ‘attack’ + ‘human’. Because some attacks recurred repeatedly during the search, due to the use of multiple sources, we cross-checked attack location, date, and sex/age of the people involved to avoid duplicate records in the dataset (additional information on the data collection method is available in^2^).

For each attack, we recorded the following information: (1) year; (2) month; (3) location of the attack; (4) time of day, classified into three categories, i.e. twilight, day and night; (5) sex and age (subadult, <4 years old; adult, >4 years old) of the bear; (6) sex and age of the victim^21^, where age was classified into two categories (child, <13 years old; adult, >13 years old); (7) human party composition, simplified into four categories^33^: (a) adult alone, (b) child alone, (c) adult in a group, and (d) child in a group; (8) result of the attack, i.e., injury or death; (9) human activity at the time of the attack, three categories: (a) leisure activities, e.g., hiking, camping, fishing, berry/mushroom/antler picking, (b) hunting and (c) outdoor work, e.g., guarding livestock, farming, logging, wildlife-related fieldworks; and (10) attack scenario, i.e., the main reason that could have triggered the attack. We defined five different scenarios: (a) attack by a female with cubs; (b) sudden encounter with a solitary bear, i.e., when a bear (except females with cubs) was surprised at a close distance; (c) predatory, i.e., when the bear deliberately attacked and/or killed a human with the presumed purpose of consuming it^26^; (d) dog-related scenario, i.e., one or more dogs were present at the moment of the attack; (e) wounded bear (i.e. a bear that was shot or trapped during hunting).

#### Information related to the countries of the attacks

For each country/jurisdiction where an attack occurred (excluding Russia, for which we lacked information), we recorded: (a) bear density, i.e., bears per 1000 km^2^; (b) human population density, i.e., inhabitants per km^2^, within the range occupied by brown bears; and (c) brown bear management practices. Specifically, we were interested in exploring possible differences in attack patterns between countries where the brown bear is legally hunted or harvested and countries where bear hunting is forbidden. We therefore classified each country/jurisdiction as either a ‘hunting country’ (i.e., where the brown bear is legally hunted or where the species is protected, but legally and regularly harvested) or a ‘non-hunting country’ (i.e., where brown bear is protected and killing them is generally forbidden).

Estimates of bear and human population densities and bear ranges for Europe and Turkey were obtained from^15,43^ and^44^. The numbers of bears for North American jurisdictions were obtained from published literature and reports^45–50^. Bear ranges and human population densities for these jurisdictions were calculated in QGIS^51^. Specifically, we derived bear ranges and human population densities using the North American border layer^52^, the IUCN layer of bear distribution^53^ and the gridded world population data set^54^. Bear ranges included areas of both permanent and occasional brown bear presence. All the above-mentioned values for each country/jurisdiction are reported in Table 1.

### Data analysis

#### Spatio-temporal patterns of the attacks

To explore general spatio-temporal patterns of bear attacks, we built a set of a priori competing models that included the number of attacks as the response variable. As explanatory variables, we included: (1) year of the attack; (2) ‘hunting’ or ‘non-hunting’ country; and (3) continent, i.e., North America and Europe. We did not include the Eastern part of bear distribution in these analyses, given the spatial heterogeneity of the data, as we were able to only collect attack data from Iran, Turkey and some Russian areas. Fixed factors also included (4) bear density and (5) human population density. To test for potential interactions between variables, we additionally included: (6) the interaction between bear density and human density; (7) the interaction between continent and bear density; and (8) the interaction between continent and human density. Correlation between explanatory variables was calculated before building the models and was always lower than 0.6. Moreover, because we had repeated measures throughout the dataset, we included country as a random factor. Following preliminary data exploration and model diagnostics, we excluded two years of data for Romania (i.e., 21 attacks in 2014 and 17 attacks in 2015) and all observations from Croatia (i.e., 3 attacks in total), which were found to be highly influential observations. Consequently, our model was run on a sample size of 431 attacks. All models were fitted using GLMMs with a Poisson distribution.

We selected the best model(s) based on AICc values and we considered models with ΔAICc < 2 as equally competitive. Once we obtained the best set of models, we selected the most parsimonious (i.e., the model that included the lowest number of explanatory variables) and, for this model, we estimated fitted parameters, confidence intervals (CI), as well as the variance explained by each explanatory variable. All statistical analyses were performed in R v. 3.5.1 statistical software^55^. Models were fitted using the “glmer” function from the “lme4” package^56^. Model generation and AICc calculation were done using the “dredge” function from the “MuMIn” package^57^. Calculation of R-squared for the model and each explanatory variable was done using the “r2glmm” package^58^.

## Supplementary information

Supplementary Information
