# Supplementary material for: Brown bear attacks on humans: a worldwide perspective
Source: Sci Rep. 2019 Jun 12;9:8573. doi: 10.1038/s41598-019-44341-w (PMC6562097; doi:10.1038/s41598-019-44341-w)
Supplement: Supplementary file 1 — Supplementary Information [file 41598_2019_44341_MOESM1_ESM.docx]

**Supplementary Information**

**Brown bear attacks on humans: a worldwide perspective**

G. Bombieri^1, 2¶*^, J. Naves^3¶^, V. Penteriani^1,4^, N. Selva^5^, A. Fernández-Gil^3^, J.V. López-Bao^1^, H. Ambarli^6^, C. Bautista^5^, T. Bespalova^7^, V. Bobrov^8^, V. Bolshakov^9^, S. Bondarchuk^10^, J.J. Camarra^11^, S. Chiriac^12^, P. Ciucci^13^, A. Dutsov^14^, I. Dykyy^15^, J. M. Fedriani^16^ , A. García-Rodríguez^5^, P. J. Garrote^16^, S. Gashev^17^, C. Groff^18^, B. Gutleb^19^, M. Haring^20^, S. Härkönen^21^, D. Huber^22^, M. Kaboli^23^, Y. Kalinkin^24^, A. A. Karamanlidis^25^, V. Karpin^26^, V. Kastrikin^27^, L. Khlyap^8^, P. Khoetsky^28^, I. Kojola^29^, Y. Kozlow^30^, A. Korolev^31^, N. Korytin^9^, V. Kozsheechkin^32^, M. Krofel^33^, J. Kurhinen^26,34^, I. Kuznetsova*^9^*, E. Larin*^7^*, A. Levykh*^17^*, V. Mamontov^35^, P. Männil^36^, D. Melovski^37,38^, Y. Mertzanis^39^, A. Meydus^40^, A. Mohammadi^41^, H. Norberg^42^, S. Palazón^43^, L.M. Pătrașcu^44^, K. Pavlova^45^, P. Pedrini^2^, P.Y. Quenette^11^, E. Revilla^3^, R. Rigg^20^, Y. Rozhkov^46^, L. F. Russo^1^, A. Rykov^47^, L. Saburova^35^, V. Sahlén^48^, A. P. Saveljev^30^, I. V. Seryodkin^49,50^, A. Shelekhov^51^, A. Shishikin^52^, M. Shkvyria^53^, V. Sidorovich^54^, V. Sopin^40^, O. Støen^55,56^, J. Stofik^57^, J. E. Swenson^55,56^, D. Tirski^45^, A. Vasin^58^, P. Wabakken^59^, L. Yarushina^7^, T. Zwijacz-Kozica^60^ & M.M. Delgado^1^.

**Supplementary Table S1.** Comparison of the competing models built to analyse the spatial-temporal patterns of brown bear attacks on humans between 2000 and 2015 based on values of AICc, ΔAICc and AICc weights. Only the models with ΔAICc < 2 are shown. Competitive models are ranked from the lowest to the highest AICc value. Response variable: number of attacks – Poisson distribution error. R^2^ = 0.427.

| ***COMPETING MODELS*** |  | ***AICc*** | ***ΔAICc*** | ***AICc weights*** |
| --- | --- | --- | --- | --- |
| *HUNTING COUNTRY+BEAR DENSITY+HUMAN DENSITY+YEAR* |  | *600.3* |  | *0.21* |
| *HUNTING COUNTRY +BEAR DENSITY+HUMAN DENSITY+YEAR+BEAR DENSITY:HUMAN DENSITY* |  | *601.2* | *0.98* | *0.13* |
| *BEAR DENSITY+HUMAN DENSITY+ YEAR* |  | *601.3* | *1.06* | *0.12* |
| *CONTINENT+HUNTING COUNTRY+BEAR DENSITY+HUMAN DENSITY+YEAR* |  | *602.2* | *1.91* | *0.08* |

**Supplementary Table S2.** Summary of fitted parameters for the most parsimonious model within the best set of models (models with ΔAICc < 2 shown in Supplementary Table S1) built to analyse the spatial-temporal patterns of the attacks by brown bears on humans between 2000 and 2015. Estimate (β), standard error (SE), p-value (p), confidence interval (CI) and the variance explained are shown for each explanatory variable. R^2^ = 0.468.

| ***EXPLANATORY VARIABLE*** | ***β*** | ***SE*** | ***p*** | ***CI*** | ***VARIANCE EXPLAINED*** |
| --- | --- | --- | --- | --- | --- |
| *INTERCEPT* | *0.758* | *0.055* | *< 0.0001* | *(0.616; 0.863)* |  |
| *BEAR DENSITY* | *0.663* | *0.066* | *< 0.0001* | *(0.474; 0.793)* | *0.389* |
| *HUMAN DENSITY* | *-0.352* | *0.078* | *< 0.0001* | *(-0.510; -0.139)* | *0.118* |
| *YEAR* | *0.222* | *0.052* | *< 0.0001* | *(0.122; 0.325)* | *0.133* |

**
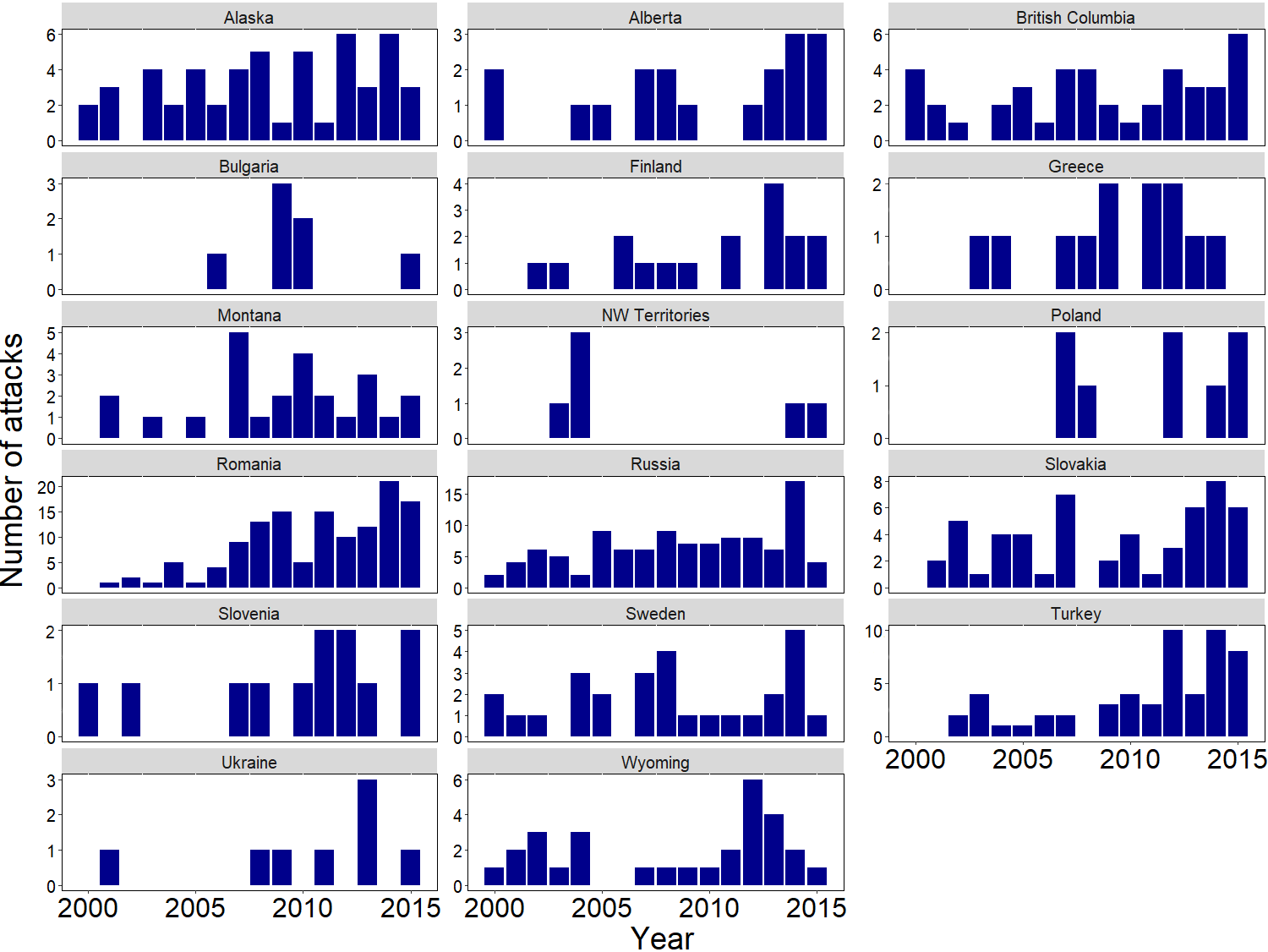
Supplementary Figure S1.** Temporal trends of brown bears attacks on humans presented for each country/jurisdiction under study between 2000 and 2015. Only countries/jurisdictions with more than five attacks are reported.

**Supplementary Figure S2.** Seasonal (**a**; n = 621) and circadian (**b**; n = 416) patterns of the attacks by brown bears on humans between 2000 and 2015.

**
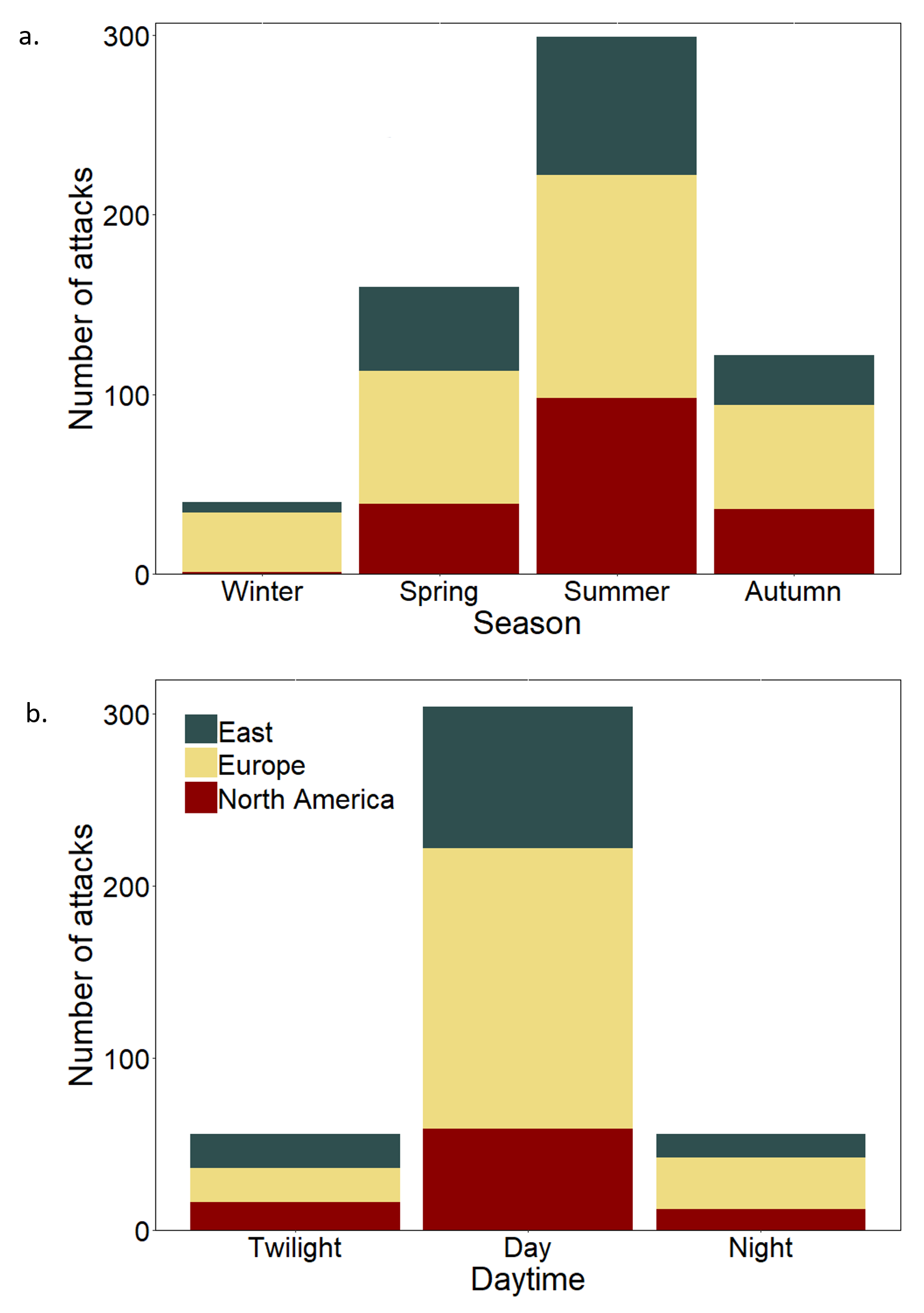
**

**
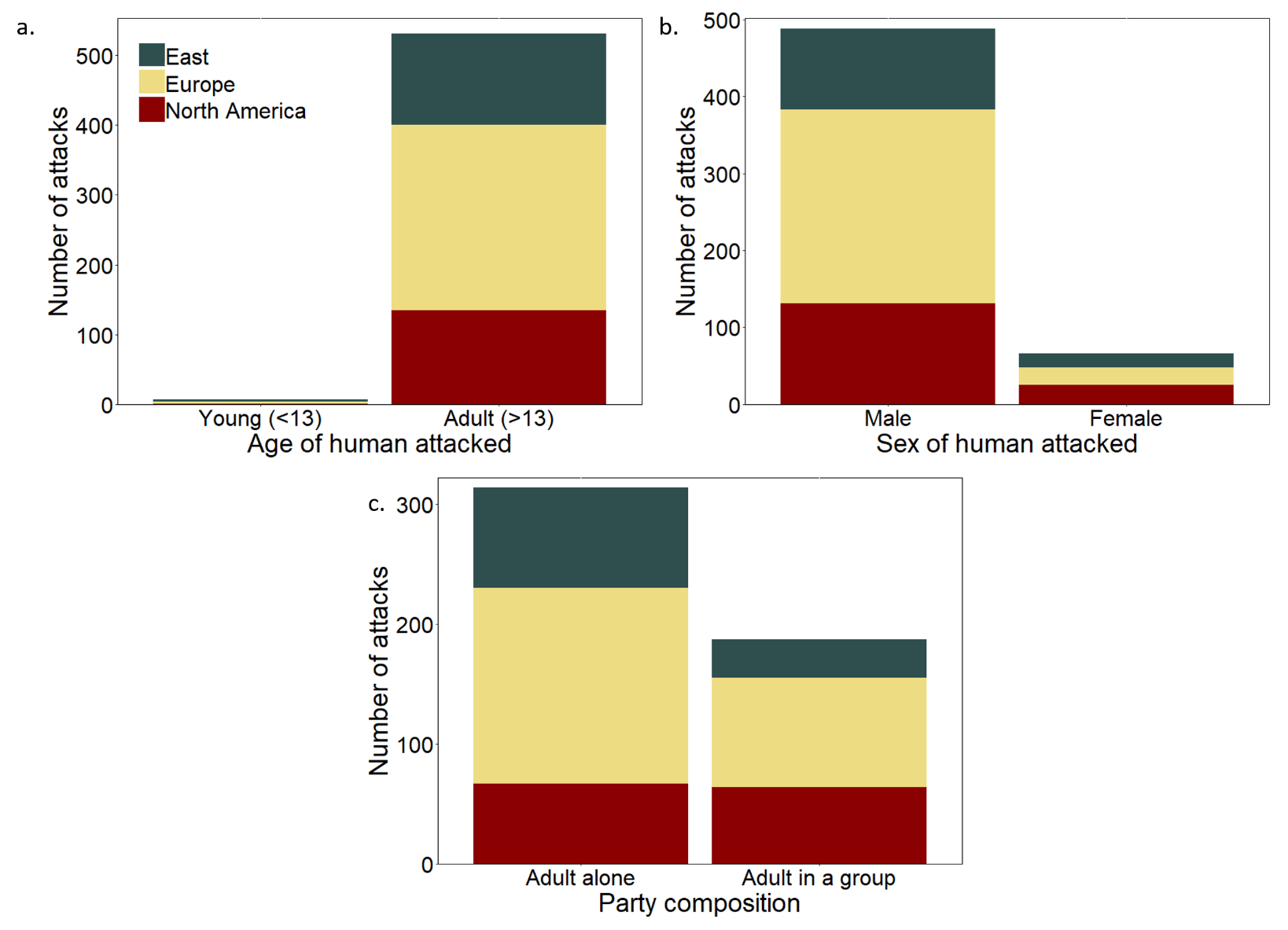
Supplementary Figure S3.** Characteristics of humans attacked by brown bears between 2000 and 2015: age (**a**; n = 538), sex (**b**; n = 554), group size and composition (**c**; n = 501).
